# Change in oral hygiene behaviour after non‐surgical periodontal therapy – A retrospective analyses

**DOI:** 10.1111/idh.12593

**Published:** 2022-04-11

**Authors:** Therese A. Elkerbout, Dagmar Else Slot, Mirella E. Rijnen, G. A. (Fridus) van der Weijden

**Affiliations:** ^1^ Department of Periodontology Academic Centre for Dentistry Amsterdam (ACTA), a joint venture between the Faculty of Dentistry of the University of Amsterdam and the Faculty of Dentistry of the Vrije Universiteit Amsterdam Amsterdam The Netherlands; ^2^ Clinic for Periodontology Utrecht Utrecht The Netherlands

**Keywords:** oral health behaviour, oral health promotion, oral hygiene behaviour, periodontal therapy

## Abstract

**Aim:**

This retrospective analysis investigates changes in daily oral hygiene behaviour after the initial phase of non‐surgical periodontal therapy (NSPT).

**Materials and Methods:**

This retrospective study includes 189 consecutive periodontitis patients treated for moderate to severe periodontitis. The authors used the oral hygiene behaviour questionnaire (OHB‐9) to assess and evaluate the oral hygiene self‐care practices at both intake and evaluation after an active phase of NSPT which consisted of repeated oral hygiene instruction (OHI), supra‐ and subgingival debridement and polishing. In addition, data on pocket probing depth and bleeding upon pocket probing (BOP) were extracted and PISA and PESA scores were collected. All these parameters are descriptive of the periodontal status at intake and the clinical response to NSPT.

**Results:**

The OHB‐9 showed an increased oral hygiene self‐care level after the active phase of NSPT. At the evaluation, 85% of patients used a power toothbrush (PTB), representing an increase of 26% as compared with the intake. In addition, 64% reported brushing 3 min or longer, representing an increase of 33%. The use of woodsticks (WS) and interdental brushes (IDB) at least once a day increased with 15% and 40% respectively. The distribution changes on the answering scale were significant for IDB, WS and brushing duration. However, no significant relationship was found between oral hygiene behaviour at the evaluation and the improvement of the gingival inflammation evaluated by BOP percentage.

**Conclusion:**

The finding of the OHB‐9 questionnaire reported was an increase of patients who brushed longer and used the PTB, WS and IDB on a daily basis. The observed improvements in gingival inflammation assessed as bleeding on probing tendency were not significantly associated with oral hygiene behaviour at the evaluation of NSPT.

## 1 INTRODUCTION

Periodontitis is an inflammatory disease of the supporting structures of the teeth mediated by the interaction between the host and the oral microbiome.^1^ Symptoms associated with periodontal disease can be halitosis, swollen or receding gingiva and tooth mobility. Periodontitis is considered to account for 30%–35% of all tooth loss.^2^ This directly affects quality of life in terms of reduced functional capacity (e.g. chewing or biting), self‐esteem and social relationships.^3^, ^4^, ^5^ The goal of periodontal therapy is to preserve, improve and maintain natural dentition.^5^ The foundation of effective periodontal therapy is mechanical debridement of the root surface, with the intention of disrupting the established biofilm. Moreover, the oral hygiene instructions (OHI) as part of the periodontal therapy induce a behavioural change in patients to improve the level of self‐performed daily oral hygiene.^6^ It is suggested that the impact of change in oral hygiene behaviour has an effect on plaque and bleeding scores. The maintenance of periodontal health following active periodontal therapy involves lifelong supportive care that consists of daily removal of the biofilm by the patient.^7^

Regarding the frequency of daily oral hygiene, both the European Federation of Periodontology (EFP) and the American Dental Association (ADA) recommend that people should brush their teeth twice a day for at least 2 min using a fluoride‐containing toothpaste. Furthermore, they advise performing a thorough daily interdental cleaning.^8^, ^9^ Regarding interdental cleaning, a systematic review (SR) from the XIII European workshop on Periodontology has suggested that interdental cleaning with interdental brushes (IDBs) is the most effective method for interdental plaque removal.^9^, ^10^ The majority of the included studies have failed to demonstrate that flossing is generally effective in plaque removal. Worthington et al.^11^ have also shown that IDBs are likely more effective than dental floss. This is supported by two Bayesian network meta‐analyses that quantitatively evaluate interdental oral hygiene devices and provide a global ranking of their efficacy.^12^, ^13^ Woodsticks and floss ranked last,^14^ and IDBs and oral irrigator ranked highest for the reduction of gingival bleeding. Moreover, in periodontal maintenance patients, a recent network meta‐analysis has demonstrated that the adjuvant use of IDBs was significantly more effective for plaque removal than using the manual toothbrush alone.^12^

To evaluate the impact of personalized OHI and guidance, the oral hygiene behaviour (OHB) questionnaire has been proposed.^15^, ^16^ The questionnaire appears to be a useful method for assessing and evaluating the oral self‐care practices of individuals.^16^ Details of toothbrushing and other potential components of personal oral hygiene are included in the OHB questionnaire, such as the use of woodsticks, interdental brushes, toothpaste with or without fluoride and tongue cleaning.^17^ So far, the OHB questionnaire has been used to evaluate oral hygiene behaviour in several populations across different continents,^18^, ^19^, ^20^, ^21^ it has not yet been applied to periodontal therapy.

The aim of the present retrospective analysis was to assess changes in patient daily oral hygiene behaviour at both intake and at evaluation after non‐surgical periodontal therapy (NSPT) with the aid of the OHB questionnaire. Moreover, this study aimed to assess whether there is a relationship between oral hygiene behaviour and changes in gingival inflammation displayed in a BOP percentage.

## 2 MATERIAL AND METHODS

### 2.1 Study design

This retrospective analysis was prepared according to the guidelines suggested by The Strengthening the Reporting of Observational Studies in Epidemiology (STROBE) Statement^22^, ^23^ and RECORD checklist.^24^, ^25^ These checklists recommend items that should be included in the reports of observational studies and studies that use routinely collected observational data. The patients in this study provided signed, informed consent in advance for the anonymous use of data related to their treatment. The institutional review board of the Academic Centre for Dentistry Amsterdam (ACTA) provided approval for this study under number (#202019).

### 2.2 Patients and OHB‐9

Patients involved in this retrospective analysis had been referred by their general dentist to the specialist Clinic for Periodontology, Utrecht due to moderate to severe periodontitis. They were treated within 12 months following their intake appointment. Prior to their intake appointment, all patients received the Dutch version of the OHB survey questionnaire, which is part of the regular procedure at the clinic, at their individual home addresses. Patients were requested to bring the completed questionnaire to their first appointment. The original OHB^16^ with eight questions was modified to nine questions (OHB‐9) as previously presented by Al‐Maliky et al.^20^ and Van Gils et al.^21^ The additional question inquiries about the type of toothbrush the patients use: a manual toothbrush (MTB), a power toothbrush (PTB) or both, the so‐called hybrid brushers. A hybrid brusher can, for example, be someone who is using a MTB in the morning and a PTB in the evening.^21^, ^26^

The OHB‐9^20^, ^21^ questionnaire includes the following nine questions:

1. Frequency of toothbrushing
2. Moments of toothbrushing
3. Measure of force of toothbrushing
4. Time of brushing
5. Toothpaste with/without fluoride
6. Type and frequency of Interdental cleaning device
7. Tongue cleaning
8. Type of toothbrush
9. Brushing method

### 2.3 Clinical measurements and treatment

The intake appointment included an intra‐ and extra‐oral assessment and a full‐mouth periodontal charting with a complete set of radiographs (for further details, see Van der Weijden et al.).^27^ In brief, the assessed periodontal parameters include the number of teeth, pocket probing depth (PPD), with measurements rounded off to the nearest millimetre and bleeding on probing (BOP), scored as absent or present. PPD and BOP were recorded at six sites (mesio‐buccal, buccal, disto‐buccal, mesio‐lingual, lingual and disto‐lingual). The dental software program calculated, based on these collected data, the periodontal inflamed surface area (PISA) and periodontal epithelial surface area (PESA) at six sites per tooth.^28^, ^29^, ^30^

Following the intake appointment, the active phase of NSPT began. Patients received two to five 1‐h sessions of thorough, non‐surgical, supra‐ and subgingival scaling and root planing. These sessions were provided by a dental hygienist primarily with ultrasonic instruments complemented with manual instruments under local anaesthesia, as indicated by the individual needs of the patient. Elective systemic antimicrobial medication consisting of a combination of amoxicillin (375 mg) and metronidazole (250 mg) was provided after the treatment sessions at the indication of the periodontist responsible for the treatment. At every session, each patient received personalized OHI from the dental hygienist to motivate and encourage them to obtain a high standard of self‐performed plaque control.^27^ The basic oral hygiene recommendations of the Clinic for Periodontology are as follows: use a PTB with a fluoride toothpaste twice a day and use interdental brushes once a day with the different sizes specified to be the best suited per interdental area. The patients returned for evaluation approximately 2.5 months after the last NSPT session. Prior to this appointment, they again filled out the OHB‐9 questionnaire. The same periodontist then again assessed the clinical parameters in the same way as described above for the baseline measurements (see Appendix).

### 2.4 Data extraction and analysis

The treatment coordinator (MER) at the clinic retrospectively extracted all data from the treatment records and anonymously entered them into an excel file. For the analysis, data were uploaded in IBM SPSS Statistics for Windows, Version 27.0.^31^ The software presents age at intake and the clinical parameters of interest at intake and evaluation are presented as mean and standard deviation (SD). It also calculates frequencies for gender, number of teeth (with PPD >5 mm) and the distribution among the answering scale of the OHB‐9 questions at intake and at evaluation after NSPT. The analyses of the self‐assessed scale of brushing force are summarized on a 4‐point scale as suggested by Buunk‐Werkhoven et al.^15^

Chi‐square test was used for the statistical analysis of the distribution changes on the answering scale of the OHB‐9 questions. It was “a priori” decided that if a statistically significant difference occurs on the distribution of the answering scale of OHB‐9 question, further statistical analysis at a patient level would be performed. Another Chi‐square test was used to analyse transitions at the patient level from the intake to evaluation after NSPT. If individual cells on the answering scale held <10 outcomes, the Fisher's exact test was employed. In addition, a subgroup analysis was performed by combining outcome options of the OHB questions and consequently dichotomizing the answering scale. To analyse changes from intake to evaluation, a paired‐sample *t*‐test was used for clinical parameters of interest. This work assesses the relationship between a summary of oral hygiene behaviour at the evaluation appointment and changes in BOP percentage according to an independent *t*‐test and a one‐way ANOVA when appropriate. *p*‐values of <0.05 are an indicator of statistical significance.

## 3 RESULTS

### 3.1 Demographics

Data of 189 consecutive patients who filled out the questionnaire were included. The number of males was 79, and the number of females was 110. The mean age was 47.87 years, with a range of 19–79 and an SD of ±12.07 years. Table 1 presents the demographic data split by gender.

**TABLE 1:** Gender and age at intake of the retrospective included patients

	Overall	Male	Female
Gender *n* (%)	189 (100%)	79 (41.8%)	110 (58.2%)
Mean age in years (SD)	47.87 (12.07)	47.52 (13.28)	48.12 (11.18)
Range (age in years)	19–79	20–79	19–71

### 3.2 Frequency distributions

Table 2 presents the frequency distributions on the answering scale for each question on the OHB‐9 questionnaire at intake and evaluation after NSPT and Chi‐square analysis for changes in the distribution. At the intake visit, 85% of the 189 patients brushed their teeth twice or more than twice a day. Furthermore, 59% used a PTB, 29% used an MTB and 12% were hybrid users. In total, 51% brushed for 2 min, and 31% brushed for 3 min or longer. In addition, 82% used a toothpaste containing fluoride, and over 40% used woodsticks (WS) and/or an IDB at least once a day. Eighteen per cent of patients cleaned their tongues on a daily basis. At the evaluation appointment after NSPT, all patients brushed at least once daily. Among the studied population, 85% of the patients used a PTB, representing an increase of 26%. As a result, there was a decrease in the use of an MTB. The distribution change on the answering scale for type of toothbrush was significant (*p* < 0.001) when comparing intake with evaluation.

**TABLE 2:** Oral hygiene behaviour (OHB‐9) questionnaire outcomes *n* = 189 evaluated at intake and evaluation after NSPT, analysed for changes in distribution of the answering scale per OHB‐9 question

Question topic (*N* = 189)	Answering scale	Outcome		
		At intake Number of patients (percentage)	At evaluation after NSPT Number of patients (percentage)	*p*‐Value^a^
1: Frequency of toothbrushing^c^	0‐Not everyday	1 (0.5)	0	0.383
	1‐Once a day	28 (14.8)	20 (10.6)	
	2‐Twice a day	133 (70.4)	145 (76.7)	
	3‐More than 2 times a day	27 (14.3)	23 (12.2)	
2: Moments of toothbrushing	0‐Morning before breakfast	79 (41.8)	70 (37.0)	NA^b^
	1‐Morning after breakfast	103 (54.5)	117 (61.9)	
	2‐Noon	20 (10.6)	20 (10.6)	
	3‐After dinner in evening	32 (16.9)	35 (18.5)	
	4‐Before going to sleep	158 (83.6)	158 (83.6)	
3: Self‐assessed measure of toothbrushing force (on a scale of 1–10, 1 = soft, 10 = rough)	1	1 (0.5)	0	0.489
	2	1 (0.5)	3 (1.6)	
	3	6 (3.2)	4 (2.1)	
	4	7 (3.7)	13 (6.9)	
	5	48 (25.4)	50 (26.5)	
	6	43 (22.8)	49 (25.9)	
	7	55 (29.1)	46 (24.3)	
	8	24 (12.7)	23 (12.2)	
	9	1 (0.5)	1 (0.5)	
	10	3 (1.6)	0	
4: Time of brushing	0‐Shorter than one minute	6 (3.2)	0	0.000^a^
	1‐One minute	27 (14.3)	3 (1.6)	
	2‐Two minutes	97 (51.3)	65 (34.4)	
	3‐Three minutes	37 (19.6)	54 (28.6)	
	4‐Longer than three minutes	22 (11.6)	67 (35.4)	
5: Toothpaste with/without Fluoride	0‐Unknown	6 (3.2)	6 (3.2)	0.451
	1‐Toothpaste with fluoride	156 (82.5)	164 (86.8)	
	2‐Toothpaste without fluoride	27 (14.3)	19 (10.1)	
6: Interdental cleaning devices:				
Floss	0‐Never	111 (58.7)	131 (69.3)	0.097
	1‐Not everyday	49 (25.9)	40 (21.2)	
	2‐Once a day	18 (9.5)	14 (7.4)	
	3‐Twice or more times a day	11 (5.8)	4 (2.1)	
Woodsticks (WS)	0‐Never	52 (27.5)	54 (28.6)	0.001^a^
	1‐Not everyday	59 (31.2)	28 (14.8)	
	2‐Once a day	44 (23.3)	65 (34.4)	
	3‐Twice or more times a day	34 (18.0)	42 (22.2)	
Interdental brush (IDB)	0‐Never	75 (39.7)	14 (7.4)	0.000^a^
	1‐Not everyday	39 (20.6)	30 (15.9)	
	2‐Once a day	56 (29.6)	101 (53.4)	
	3‐Twice or more times a day	19 (10.1)	44 (23.3)	
7: Tongue cleaning	0‐Never	88 (46.6)	84 (44.4)	0.742
	1‐Sometimes	66 (34.9)	64 (33.9)	
	2‐Everyday	35 (18.5)	41 (21.7)	
8: Type of toothbrush	0‐Manual	55 (29.1)	7 (3.7)	0.000^a^
	1‐Power	112 (59.3)	160 (84.7)	
	2‐Both/hybrid users	22 (11.6)	22 (11.6)	
9: Brushing method	0‐Horizontal movement	103 (54.5)	64 (33.9)	NA^b^
	1‐Vertical movement	82 (43.4)	63 (33.3)	
	2‐Circular movement	66 (34.9)	70 (37.0)	
	3‐Bass method	59 (31.2)	93 (49.2)	

Abbreviation: NA, not applicable.

^a^ Statistically significant difference between intake and evaluation after NSPT using the Chi‐square test.

^b^ Patients signed possible up to more than one category.

^c^ One missing value.

### 3.3 Sub‐analyses of the changes at a patient level

Based on significant distribution changes on the answering scale of the oral hygiene behaviour (OHB‐9) questionnaire between the intake and evaluation appointment further sub‐analysis was performed. Subsequently, also at a patient level, the transition for type of TB, brushing time and the frequency of use of either WS or IDB was significant (*p* < 0.0001) (Table 3). The significant change in the use of type of TB can be explained by the 55 MTB brushers at intake who then switched to hybrid brushers (*n* = 50) or solely used PTB. At the evaluation, 91% patients made the transition from an MTB to a form of PTB use. In addition, 73% of participants who were hybrid brushers at intake switched to exclusive PTB use (Table 3). Further dichotomization of the answering scale showed that the number of patients brushing for >1 min did not significantly increase as compared with ≤1 min of brushing (*p* < 0.079). However, brushing for >2 min as compared with ≤2 min significantly increased (*p* < 0.001; Table 4). The frequency of use for both WS and IDB showed a significant change (*p* < 0.001) for daily use at a patient level (Table 4). As compared with the intake results, 11 patients brushed less often and 19 more often at the evaluation. Moreover, 66% of the sample brushed twice daily on both questionnaire assessment moments. The subgroup analysis for brushing frequency showed a significant change to brushing >once daily (*p* < 0.001; Table 5). The analyses of the self‐assessed scale of brushing force showed significant changes at the evaluation regarding using less force (Table 5).

**TABLE 3:** Sub‐analysis of significant outcomes of the distribution of the answering scale of the oral hygiene behaviour (OHB‐9) questionnaire (Table 2), evaluated by the transition in number of patients per item at intake and at evaluation after NSPT

Brushing time at evaluation						
	1 min	2 min	3 min	>3 min	Total	*p*‐Value^a^
Brushing time at intake						
<1 min	1	2	1	2	6	<0.001^a^
1 min	1	17	2	7	27	
2 min	1	43	35	18	97	
3 min	0	3	12	22	37	
>3 min	0	0	4	18	22	
Total	3	65	54	67	189	

Frequency of use of the WS at evaluation						
	Never	Not everyday	Once a day	≥Twice a day	Total	*p*‐Value^a^
Frequency of use of the WS at intake						
Never	24	6	16	6	52	<0.001^a^
Not everyday	15	19	22	3	59	
Once a day	7	2	21	14	44	
≥Twice a day	8	1	6	19	34	
Total	54	28	65	42	189	

Frequency of use of the IDB at evaluation						
	Never	Not everyday	Once a day	≥Twice a day	Total	*p*‐Value^a^
Frequency of use of the IDB at intake						
Never	12	18	32	13	75	<0.001^a^
Not everyday	1	9	23	6	39	
Once a day	1	2	39	14	56	
≥Twice a day	0	1	7	11	19	
Total	14	30	101	44	189	

Type of TB at evaluation					
	MTB	PTB	Hybrid TB	Total	*p*‐Value^a^
Type of TB at intake					
MTB	5	34	16	55	<0.001^a^
PTB	1	110	1	112	
Hybrid TB users	1	16	5	22	
Total	7	160	22	189	

Abbreviations: IDB, interdental brush; TB, toothbrush; WS, woodstick.

^a^ *p*‐Value of the Pearson's Chi‐square.

**TABLE 4:** Subgroup analysis of the oral hygiene behaviour (OHB‐9) questionnaire outcomes presenting the number of patients per item at intake and at evaluation after NSPT

Brushing time at evaluation				
	≤1 min	>1 min	Total	*p*‐Value^b^
Brushing time at intake				
≤1 min	2	31	33	0.079^b^
>1 min	1	155	146	
Total	3	186	189	

Brushing time at evaluation				
	≤2 min	>2 min	Total	*p*‐Value^b^
Brushing time at intake				
≤2 min	65	65	130	<0.001^b^
>2 min	3	56	59	
Total	68	121	189	

Frequency of use of the WS at evaluation				
	<Once daily	≥Once daily	Total	*p*‐Value^a^
Frequency of use of the WS at intake				
<Once daily	64	47	111	<0.001^a^
≥Once daily	18	60	78	
Total	82	107	189	

Frequency of use of the IDB at evaluation				
	<Once daily	≥Once daily	Total	*p*‐Value^b^
Frequency of use of the IDB at intake				
<Once daily	40	74	114	<0.001^b^
≥Once daily	4	71	75	
Total	44	145	189	

Note

Outcome data are summarized in relation to a dichotomous answering scale.

Abbreviations: IDB, interdental brush; WS, woodstick.

^a^ *p*‐Value Pearson's Chi‐square.

^b^ *p*‐Value Fisher's exact.

**TABLE 5:** Subgroup analysis of the oral hygiene behaviour (OHB‐9) questionnaire outcomes presenting the number of patients per item at intake and at evaluation after NSPT

Brushing frequency at evaluation				
	≤Once daily	>Once daily	Total	*p*‐Value^b^
Brushing frequency at intake				
≤Once daily	18	11	29	<0.001^b^
>Once daily	2	157	159	
Total	20	168	188^c^	

Toothbrush force at evaluation						
	0 = soft	1 = soft/forcefully	2 = forcefully	3 = rough	Total	*p*‐Value^a^
Toothbrush force at intake						
0 = soft	3	3	1	1	8	<0.001^a^
1 = soft/forcefully	2	32	19	2	55	
2 = forcefully	2	24	61	11	98	
3 = rough	0	4	14	10	28	
Total	7	63	95	24	189	

Notes

0 = soft (1,2,3), 1 = soft/forcefully (4,5), 2 = forcefully (6,7), 3 = rough (8,9,10). The scale for brushing frequency was dichotomized according to Al‐Maliky et al.^20^ The toothbrush force was categorized according to Buunk et al.^15^, ^16^

^a^ *p*‐Value of the Pearson's Chi‐square.

^b^ *p*‐Value Fisher's exact.

^c^ One missing value.

### 3.4 Clinical parameters

The effect of NSPT was recorded through various clinical periodontal parameters (BOP, PPD >5 mm, PISA and PESA). The mean difference between the intake and evaluation for BOP percentage was 34.95 (19.58). For the total number of sites with a PPD >5 mm, the mean difference was 15.80 (16.39), and the mean differences for PISA and PESA were 10.57 (6.72) and 6.79 (4.08) respectively. All changes were clinically significant (*p* < 0.001). At the patient level, 95% had at least one pocket >5 mm at intake which then reduced to 58% after NSPT at the evaluation. This shows that 42% of the patients had pockets ≤5 mm. The mean number of pockets >5 mm at the intake was 17.70, which then reduced to 1.90 at the evaluation. Of the 4986 teeth at the intake, 67.09% had a PPD >5 mm. During NSPT, 117 teeth were extracted. At the evaluation, there were 4869 teeth remaining, of which 7.37% showed a PPD >5 mm. Corresponding at a patient level to 11.21% of sites with PPD >5 mm at intake and 1.24% of sites with PPD >5 mm at the evaluation (Table 6a,b).

**TABLE 6:** (a) The mean bleeding on probing scores (%BOP), number of pockets with pocket probing depth (PPD) ˃5 mm, periodontal inflamed surface area (PISA) and periodontal epithelial surface area (PESA) scores of the included patients on intake, evaluation and the difference between the measurement moments. (b) Frequency of patients and teeth with PPD >5 mm

Clinical parameter of interest (*N* = 189)	Intake Mean (SD)	Evaluation of NSPT Mean (SD)	Difference Evaluation – intake Mean (SD)	*p*‐Value^a^
(a)				
%BOP	47.43 (21.16)	12.49 (12.29)	34.95 (19.58)	<0.001
# Number of pockets PPD ˃5 mm	17.70 (17.61)	1.90 (3.24)	15.80 (16.39)	<0.001
PISA	13.01 (7.28)	2.44 (2.39)	10.57 (6.72)	<0.001
PESA	22.30 (5.52)	15.52 (3.20)	6.79 (4.08)	<0.001
Mean number of teeth	26.38 (2.88)	25.76 (3.24)	0.619 (1.45)	<0.001

Parameter of interest (*N* = 189)	Intake	Percentage	Evaluation	Percentage
(b)				
Number of patients with PPD ˃5 mm	180	95%	109	58%
Total number of teeth	4986	NA	4869	NA
% Of teeth with PPD ˃5 mm	3345	67.09%	359	7.37%
% Of sites with PPD per teeth ˃5 mm	NA	11.21%	NA	1.24%

^a^ *p*‐Value of paired sample *t*‐test comparing intake and evaluation after NSPT.

Table 7 shows the assessment of the relationship between oral hygiene behaviour, regarding brushing frequency, time of brushing, frequency of use WS/IDB and type of TB, at the evaluation and the change of gingival inflammation in BOP percentage. The statistical analysis of the comparison between the values at evaluation and intake was significant (*p* < 0.001) for all these subgroups. In addition, Table 7 presents the analyses of the change in BOP percentage between the subgroups of which the answering scale of the oral hygiene behaviour question was dichotomized for daily use or minutes used. No statistically significant relationship was established.

**TABLE 7:** The relation to oral hygiene behaviour at the evaluation appointment and the change of % BOP, between intake and evaluation after NSPT

Oral hygiene behaviour item at evaluation	Subgroups	Number of patients	Change in % BOP (difference between intake and evaluation) Mean (SD)	Comparison between BOP at intake and evaluation and *p*‐value^a^	*p*‐Value^b^ of comparison of change in % BOP between groups of the oral hygiene behaviour items
Frequency brushing^d^	≤Once daily	20	36.85 (19.52)	<0.001	0.96
	>Once daily	168	34.67 (19.68)	<0.001	
Time of brushing	≤1 min	3	27.33 (31.09)	<0.001	0.23
	>1 min	186	35.07 (19.45)	<0.001	
	≤2 min	68	35.44 (19.86)	<0.001	0.71
	>2 min	121	34.67 (19.50)	<0.001	
Frequency of use of the WS	<Once daily	82	35.16 (21.38)	<0.001	0.16
	≥Once daily	107	34.79 (18.19)	<0.001	
Frequency of use of the IDB	<Once daily	44	35.91 (20.89)	<0.001	0.17
	≥Once daily	145	34.66 (19.24)	<0.001	
Type of TB	MTB	7	30.86 (24.74)	<0.001	0.44^c^
	PTB	160	35.39 (19.49)	<0.001	
	Hybrid	22	33.00 (19.26)	<0.001	

Note

Statistical analysis of the comparison between evaluation and intake values. In addition, the analyses of BOP change between the groups of oral hygiene behaviour questions. *N* = 189.

Abbreviations: IDB, interdental brush; TB, toothbrush; WS, woodstick.

^a^ Paired sample *t*‐test.

^b^ Independent *t*‐test.

^c^ One‐Way ANOVA.

^d^ One missing value.

## 4 DISCUSSION

### 4.1 Summary of findings

The purpose of this retrospective analysis was to evaluate the change in daily oral hygiene behaviour by the OHB‐9 questionnaire after an active phase of NSPT. At the selected evaluation appointment, 189 patients who had filled out the questionnaire were included, which showed that in total 85% solely used the PTB and 12% brushed alternatively with a MTB and PTB. Seventy‐seven per cent of the patients used an IDB at least once a day. This is a 26% increase in the use of a PTB, a 15% increase for the daily use of WS and a 37% increase for the daily use of IDBs. The present findings show that the OHB‐9 questionnaire can be a useful tool for analysing changes in OHB both at individual and practice‐based levels. The OHB‐9 can help oral care practices to audit the impact of treatment and OHI offered to their patients. It is an easy tool to implement and can be used to evaluate patient oral hygiene behaviour experiences. The OHB‐9^9^, ^16^ questionnaire was developed based on the OHB‐8 questionnaire.^20^, ^21^ It is shown via a Delphi method involving expert oral health professionals, that given the relatively low number of items and the substantial variety in the content of the questions, the questionnaire has a sufficient internal structure, as was apparent from its face validity.^16^

The analysis of the individual aspects on oral hygiene behaviour, such as frequency of brushing, WS and IDB, time of brushing and type of toothbrush used, found no significant relationship with the change in BOP percentage at the evaluation (Table 7). The effect of non‐surgical periodontal therapy by scaling and root planing is in general of such a high magnitude that it appears difficult to show an additional effect beyond this treatment response itself,^32^ for instance, by the use oral hygiene devices. Moreover, in an earlier systematic review, the impact of change in oral hygiene behaviour on plaque and bleeding scores was not established in patients with periodontal diseases.^33^ An alternative explanation may be that the skewed distribution of the evaluated aspect of oral hygiene behaviour within this treated periodontitis patient population prevents an association from emerging.

### 4.2 Justification of the oral hygiene recommendations

Regarding the frequency of daily oral hygiene, both the European Federation of Periodontology (EFP) and the American Dental Association (ADA) recommend that people should brush their teeth twice a day for at least 2 min using a fluoride‐containing toothpaste. Furthermore, they advise performing a thorough daily interdental cleaning.^8^, ^9^ Regarding the choice of toothbrush, many studies and SRs have concluded that rechargeable PTBs are preferable for gingivitis patients, as they are more effective than MTBs.^5^, ^9^, ^34^ PTBs with various mechanical motions and features are widely available. Instead of forcing people to concentrate on the right brushing technique, these brushes’ built‐in motions allow them to pay more attention to the right placement of the brush.^12^ A previous study has also shown that recommending a PTB for periodontitis patients showing low compliance with oral hygiene is worthwhile.^35^ A recent SR has evaluated the effects of various mechanical oral hygiene devices for periodontal maintenance patients, and it has found no evidence in support of a difference between MTBs and PTBs.^12^ This was evident from both the descriptive analysis as well as from the network meta‐analysis (NMA). This has probably to do with the fact that in periodontitis patients it is difficult to filter out only the effect of a toothbrush, as it would be unethical to abstain from interdental oral hygiene in this particular patient population. Based on the same study, when the effect of interdental cleaning devices was evaluated in periodontal maintenance patients, the additional use of IDBs reduced plaque scores most.^12^ For the reduction of gingival inflammation, no product ranked higher than the manual toothbrush.^12^

### 4.3 Oral hygiene instructions

The evidence suggests that the largest decrease in the number of periodontally diseased sites is seen following OHI and NSPT. Good oral hygiene and low plaque levels produce the best outcomes. OHI is, therefore, an integral part of NSPT.^9^, ^36^ A recent publication has observed that a statistically significant and profound reduction in plaque, BOP and PPD can be observed even after a professional OHI on its own.^37^

An SR of the available literature has argued that a single OHI combined with a single professional oral prophylaxis provides a small but significant positive effect on the reduction of gingivitis. Repeated OHIs have shown even further improvements on gingival health.^38^ Professional instructions on a personalized individual basis have also been effective at inducing a change in the efficacy of OHB.^30^ There is, however, insufficient evidence to recommend any specific, one‐to‐one OHI method as effective in improving oral health or being more effective than any other method.^17^ It has been shown that written OHIs do not adequately influence oral hygiene selfcare.^39^, ^40^ Interestingly dental hygienists also provide more detailed information about oral hygiene aids as compared with dentists.^41^

In the light of this, it is remarkable that there was one person at the intake who reported not to brush every day and 15% which brushed only once a day. Two and a half months after the NSPT and repeated OHIs, this result changed to the point that 87% brushed at least twice a day and 85% used a fluoride TP, which aligns with the advice of the ADA.^8^ Repeated OHIs are especially important because there is evidence showing that the reinforcement of oral hygiene provides further benefits.^9^

### 4.4 Success of NSPT

A recent publication has evaluated the results of NSPT in 1182 patients diagnosed with adult periodontitis. They were treated in the same specialized clinic for periodontology in Utrecht the Netherlands and had received detailed instructions on how to clean their teeth. For this patient group, success of periodontal therapy was defined as having no pockets deeper than 5 mm, and 39% of the patients reached this successful treatment objective with a mean bleeding on probing score of 14%.^27^ Applying the same success criteria to the present patient population reveals that 42% of the patients reached pocket probing depth of ≤5 mm at the evaluation visit. The mean bleeding score reduced from 47% at intake, with 35%–12% after NSPT. These results align with the previous study.

### 4.5 Brushing duration and frequency

In a previous study, brushing duration has been seen as an important variable in the efficacy of plaque removal.^39^ Slot et al.^42^ have shown that the plaque reduction efficacy of MTBs increased when the brushing time increased. Rosema et al.^34^ note the same effect for PTBs. An increase in brushing duration has also been associated with higher plaque score reduction.^34^, ^39^ In the present population, 82% reported brushing their teeth for 2 min or longer at the intake and this increased up to 98% at the evaluation. However, few studies have addressed toothbrushing frequency as a proxy for oral hygiene and periodontitis. Zimmerman et al.,^43^ for instance, has shown a relatively small but significant relationship of infrequent toothbrushing with severe forms of periodontal disease. Moreover, an earlier study by Lang et al. has shown that complete plaque removal once every 48 h is compatible with gingival health in periodontally healthy subjects.^44^ In general, it is questioned if 100% plaque removal is practically feasible. A more recent publication, however, has found that frequencies of mechanical plaque removal up to 24 h may prevent an increase in the severity of gingival inflammation in patients with no history of periodontitis, frequencies of mechanical removal plaque up to 24 h may prevent an increase in the severity of gingival inflammation.^45^ In elderly subjects, it has been found that twice daily toothbrushing contributes to better periodontal health.^46^ Ganss et al.^47^ have shown that the majority (79.6%) of uninstructed adults brushed twice daily. Higher toothbrushing frequency, however, does not necessarily ensure more efficient toothbrushing, even if a positive correlation seems likely.^43^

### 4.6 Generalizability

Previous research has shown that education, socioeconomic status (SES), race and ethnicity and general health status are significant predictors of OHB.^48^ Education can influence both OHB and even the results of the patients’ oral cleaning habits.^49^ In the present retrospective analysis, no data were available regarding these predictors so that it was not possible to specify the results with more details in this respect. The results from this study are, however, representative of a practice‐based periodontal situation in Western society.

### 4.7 Limitations

This retrospective analysis of OHB showed a positive response to repeated professional OHIs, although the evaluation time of approximately 2.5 months following completion of the active phase of NSPT was short. One possibility is that the behavioural change as observed is short‐lived. The dedication that is required to maintain a high degree of plaque control may deteriorate rapidly given that improved gingival health tends to fade over time and return to its original value.^30^, ^40^ There is also evidence showing that the reinforcement of oral hygiene provides further benefits.^9^ Patients from the present analysis will continue in a periodontal maintenance program which includes regular OH evaluation and if needed individual instruction. This will support them to preserve the improvement in OHB.

Given that this retrospective analysis involved a group of patients who were treated in a clinic specialized and restricted to periodontal therapy, this patient group may be more motivated and interested in maintaining oral health. This may have an impact on the observed changes in OHB.^3^

As this retrospective analysis included patient‐related data gathered before the introduction of the new classification of periodontal disease,^50^ the diagnosis of moderate to severe adult periodontitis was based on the classification suggested by Van der Velden.^51^, ^52^

Due to the retrospective nature of the study design, there was no examiner calibration for BOP and PPD measures.

No plaque index scores were collected. However, at the beginning of each appointment with the dental hygienist, plaque was disclosed and subsequent individualized hands‐on instructions were given.

Although considered rational, the additional question to the OHB‐8 with respect to the type of toothbrush used was not part of the validation process.

In general, people overestimate their oral hygiene efforts or state what they believe the dental care professionals would like to hear.^53^

### 4.8 Suggestion for further research

This study used a questionnaire that was previously validated. It is of interest to evaluate after NSPT including an intensive aspect of personalized oral hygiene instructions and guidance what the effect is on details of the toothbrush and interdental cleaning brands and type. In addition, the real time of use, instead of the categories given for the questionnaire.

The OHB‐9 questionnaire evaluated the change of use, but no information could be retrieved whether this was based on an individual specific recommendation. It would be worthwhile to assess patient behaviour and compliance in relation to professional recommendations.

## 5 CONCLUSION

The findings of the OHB‐9 questionnaire show that at evaluation after an active phase of NSPT, there was an increase in patients who brushed longer and used the PTB, WS and IDB on a daily basis. The observed improvements in gingival inflammation assessed as bleeding on probing tendency were not significantly associated with oral hygiene behaviour at the evaluation of NSPT.

## 6 CLINICAL RELEVANCE

### 6.1 Scientific rationale

Dental plaque leads to gingivitis and can eventually turn into periodontitis. Adequate and self‐performed daily oral hygiene is essential for maintaining oral health.

### 6.2 Principal findings

Following a non‐surgical periodontal therapy phase with repeated professional personalized oral hygiene instruction, this retrospective analysis shows that patients used the power toothbrush, woodsticks and interdental cleaning devices more often.

### 6.3 Practical implications

The oral hygiene behaviour questionnaire (OHB‐9) is a useful method for assessing and evaluating oral hygiene self‐care practices and provides reflections regarding changes and improvements in oral hygiene self‐care.

## CONFLICT OF INTEREST

The authors declare that they have no conflicts of interest.

## AUTHOR CONTRIBUTIONS

All authors gave final approval and agreed to be accountable for all aspects of work ensuring integrity and accuracy. TAE contributed to design, analysis and interpretation, and drafted the manuscript. DES contributed to data analysis and interpretation and critically revised the manuscript. MER contributed to design and data collection. GAW contributed to conception and design, analysis and interpretation and critically revised the manuscript.

## Data Availability

The data that support the findings of this study are available from the corresponding author upon reasonable request.

## Appendix

Flow chart of the patient flow of the retrospective analysis.
